# Inhibition of excessive autophagy and mitophagy mediates neuroprotective effects of URB597 against chronic cerebral hypoperfusion

**DOI:** 10.1038/s41419-018-0755-y

**Published:** 2018-06-28

**Authors:** Shao-Hua Su, Yi-Fang Wu, Da-Peng Wang, Jian Hai

**Affiliations:** Department of Neurosurgery, Tongji Hospital, Tongji University School of Medicine, 200065 Shanghai, China

## Abstract

URB597 (URB) has therapeutic potential for treating chronic cerebral hypoperfusion (CCH)-induced neuronal death. The present study investigated the protective effects of URB on autopahgy and mitophagy in a CCH model as well as the underlying mechanisms. The ultrastructural changes were examined by electron microscopy. The mitochondrial membrane potential was assessed by immunofluorescence. The expressions of autophagy-related proteins (beclin-1, p62, and LC3), lysosome-related proteins (CTSD and LAMP1), and mitophagy-related proteins (BNIP3, cyt C and parkin) were evaluated by western blotting, and the interaction of beclin-1 and Bcl-2 were determined by immunoprecipitation. CCH significantly decreased the protein expression of p62, CTSD, and LAMP1 and increased the protein expression of beclin-1, parkin, and BNIP3, the LC3-II to LC3-I ratio, and the release of cyt C from mitochondria to cytoplasm. Furthermore, CCH induced the accumulation of ubiquitinated proteins in PSDs. However, URB significantly reversed these results. Besides, URB significantly inhibited the beclin-1 from beclin-1/Bcl-2 complex to whole-cell lysates. The above results indicate that URB could inhibit impaired autophagy degradation and the disruption of beclin-1/Bcl-2 complex and subsequently cut off BNIP3-cyt C- and parkin-required mitophagy, finally preventing the abnormal excessive autophagy and mitophagy. These findings provide new insights that URB is a promising agent for therapeutic management of CCH.

## Introduction

Chronic cerebral hypoperfusion (CCH), a chronic state of cerebral blood flow reduction, is a common pathological process associated with many cerebrovascular diseases including Alzheimer’s disease, artherosclerosis, carotid stenosis/occlusion, moyamoya disease, and cerebral small vessel disease^1–5^. It is an important vascular risk which contributes to neuronal apoptosis, cognitive impairment, and neuroinflammatory responses^6–8^. In the pathogenesis of CCH, the delayed apoptotic cell death is often found in brain regions which are vulnerable to cerebral hypoperfusion^9^. Furthermore, neuronal apoptosis plays a vital role in the progression of CCH-induced cognitive impairment^10^. Hence, protection against neuronal apoptosis may be a potentially effective therapeutic strategy for CCH. In our previous study^8^, we found that non-selective cannabinoid receptor agonist WIN55,212-2 (WIN) and fatty acid amide hydrolase inhibitor URB597 (URB) may exert the anti-apoptosis effects at least partially through inhibiting the JNK-dependent non-nuclear pathway. Moreover, URB may offer increased selective protection with less risk of the undesirable side effects that have been observed with cannabinoid receptor agonists capable of activating all accessible receptors indiscriminately. Targeted URB treatment is considered a more attractive therapeutic approach. However, the mechanisms behind the URB-mediated inhibition of CCH-induced neuronal damage require further clarification.

Autophagy, a process through which cells recycle cytoplasm and remove defective organelles, is essential for maintaining cellular homeostasis which requires the continual turnover of non-functional proteins and organelles^11^. Besides necrosis and apoptosis, autophagy has been identified as a third type of cell death that occurs during ischemic stroke^12^. Autophagy is reported to have controversial effects (both detrimental and beneficial) in cerebral ischemia^13–15^. In our opinion, under the condition of CCH, autophagy at low level is nessesary for the cell survival, while the excessive activated autophagy would bring serious damage to the proper environments of cell survival. However, there are little data thus far supporting this conjecture. Furthermore, the interactions between autophagy and apoptosis are complex with much crosstalk, and their roles and their pathologic processes in CCH are also not clear, which need further investigations. In addition, the possible mechanisms regarding mitophagy in CCH are not well elucidated. A better understanding of autophagy and the interactions between autophagy and apoptosis might provide new therapeutic targets for the treatment of CCH.

To address these issues, in the present study, we attempted to explore the role of autophagy and mitophagy, the underlying mechanisms of autophagy and mitophagy, and the interactions between autophagy and neuronal apoptosis under the condition of CCH as well as the treatment of URB.

## Materials and methods

### Animal administration

Male Sprague-Dawley rats, weighing 230–250 g, were purchased from the experimental animal center of Shanghai Sippr-BK Laboratory Animals Ltd. (Shanghai, China). Prior to experiments, the rats were housed for 12 weeks in climate-controlled facilities on a 12:12 h light–dark cycle (lights on from 08:30–20:30) at constant temperature (23 ± 1 °C) and humidity (60%) with free access to food and water. After 1 week of acclimatization, rats were randomly divided into six groups (5 rats per group) as follows: (1) Sham-operated; (2) Bilateral common carotid artery’s occlusion (BCCAo); (3) BCCAo+ the autophagy inhibitor 3-methyladenine (3-MA; Sigma, St. Louis, MO, USA); (4) BCCAo+c-Jun N-terminal kinase (JNK) inhibitor SP600125 (Sigma, St. Louis, MO, USA); (4) BCCAo+URB (Cayman Chemicals, Tallinn, Estonia); (5) BCCAo+URB+3-MA; (6) BCCAo+URB+SP600125. After 12 weeks, rats were sacrificed, and their brain were immediately removed for experiments or stored at −70 °C for later use.

Experimental protocols were performed in compliance with international guidelines and Chinese legislation on the use and care of laboratory animals, and were approved by the Animal Laboratory Center of Tongji University School of Medicine.

### Drug treatment

All the experimental drugs were administrated after BCCAo for consecutive 12 weeks. Sham-operated and BCCAo groups were daily administered an equivalent volume of vehicle solution. URB (0.3 mg/kg/day, intraperitoneal (i.p.) injection) and SP600125 (30 µg, intracerebroventricular (i.c.v.) injection) dosages were selected based on our previous studies^6–8^. Based on our pilot studies, low dose of 3-MA (1 µg) had little effect while median dose (3 µg) alleviated the autophagy induced by CCH. However, high dose (5 µg) induced excessive reduction of autophagy in 67% of rats. 3-MA was therefore administered at 3 µg/day by i.c.v. injection in our study, which is accordance with other study^16^. Guide catheters were implanted into both cerebral ventricles (distance from bregma: anteroposterior, 0.5 mm; mediolateral, 1.0 mm; depth, 2.0 mm)^17^. Daily infusion of SP600125, 3-MA, or solvent was delivered using a microinjector (KD Scientific Inc., Holliston, MA, USA) into both cerebral ventricles at a rate of 2.5 μl/min for 12 weeks according to the earlier report^7,8,18^ and previously established protocols in our laboratory.

### Surgery procedure

The maximum chronic hypoperfusion phase in a rat model of CCH by BCCAo may be 12 weeks^19^, which was used in the present study. Rats were anesthetized with sodium pentobarbital (50 mg/kg, i.p.), and a midline incision was made to expose the bilateral common carotid arteries, which were tightly double-ligated with 5-0 silk sutures. Sham-operated animals were subjected to the same procedure but without arterial ligation.

### Electron microscopy

For synaptic ultrastructural changes, tissue sections were stained with 1% ethanolic phosphotungstic acid (EPTA, Fisher Scientific, Fairlawn, NJ) as in previous study^20^. Briefly, coronal brain sections were cut to a thickness of 120 μm with a vibratome at the level of the CA1 hippocampus and were postfixed for 1 h with 4% glutaraldhyde in 0.1 M cacodylate buffer (pH 7.4). Sections were then dehydrated in an ascending ethanol series to 100% and stained for 50 min with 1% phosphotungic acid (PTA) prepared by dissolving 0.1 g PTA in 10 ml of 100% ethanol and adding 150 μl of 95% ethanol. The EPTA solution was changed once after a 15-min interval during staining. Sections were further dehydrated in dry acetone and embedded in Durcupan ACM resin. Randomly selected ultrathin sections were examined under an electron microscopy (Philips, Amsterdam, Netherlands).

For autophagosomes, according to our previous study^21^, the coronal slices in the CA1 hippocampal area approximately 1 mm thick were fixed in 2.5% glutaraldehyde overnight at 4 °C, washed by 0.1 mol/l PBS for three times, and were postfixed in 1% osmium tetroxide for 2 h at 4 °C. Then, the blocks were dehydrated in graded ethanol, and embedded in epoxy resin. Randomly selected ultrathin sections (60–70 nm) were poststained with uranyl acetate and lead citrate, and examined under an electron microscopy (Philips, Amsterdam, Netherlands).

### Isolation of mitochondria

The mitochondrial fraction was isolated using an Qproteome mitochondria isolation kit (Qiagen, Düsseldorf, GER). Briefly, 20 mg tissues were cut into pieces and homogenized in 2 ml lysis buffer with protease inhibitor solution. The supernatant (containing cytosolic proteins) was collected by centrifugation at 1000×*g* for 10 min at 4 °C. The pellet was resuspended and disrupted in 1.5 ml ice-cold disruption buffer. After centrifugation at 1000 × *g* for 10 min at 4 °C, the supernatant was collected and centrifuged at 6000 × *g* for 10 min. The pellet (containing mitochondria) was resuspended in 750 μl mitochondrial purification buffer and added on top of a mitochondrial purification buffer layer (500 μl disruption buffer under 750 μl mitochondrial purification buffer). After centrifugation at 14000 × *g* for 15 min, a pellet or band containing mitochondia was formed in the lower part of the tube, which was transferred to a new tube. The suspension was washed three times with 1.5 ml mitochondrial storage buffer by centrifugation at 8000 × *g* for 10 min. The high-purified mitochondria were resuspended in mitochondrial storage buffer. Fresh mitochondria were used for membrane potential detection. Mitochondrial and cytosolic fractions were stored at −70 °C for later use.

### Measurement of mitochondrial function

The mitochondrial function was measured using a JC-1 staining kit (Genmed Scientifics Inc., Minneapolis, MN, USA). Briefly, 10 μl of high-purity mitochondria (containing 5 μg mitochondrial proteins) were incubated in JC-1 staining reagent (100 μl) for 10 min. Images were captured using a microscope (Olympus IX71; Olympus, Tokyo, Japan) at wavelengths of 594 and 488 nm. Healthy mitochondria, which have a high potential, showed a high intensity of red fluorescence at 594 nm and a low intensity of green fluorescence at 488 nm; damaged mitochondria showed a low intensity of red fluorescence at 594 nm and a high intensity of green fluorescence at 488 nm. The ratio of red/green intensity was measured to evaluate the function of isolated mitochondria from each group.

### Western blot analysis

Samples (20 µg proteins) were separated on a 10% or 12% sodium dodecyl sulfate polyacrylamide gel and transferred to a nitrocellulose membrane, which was blocked with 5% non-fat milk and 0.1% Tween-20 in Tris-buffered saline for 1 h and then incubated overnight at 4 °C with primary antibodies against beclin-1 (1:200, Abgent), microtubule-associated protein 1 light chain 3 (LC3, 1:200, Abgent), AKT (1:2000, Cell Signaling Technology), phosphorylated (p-)AKT (Ser473, 1:2000, Cell Signaling Technology), AMP-activated protein kinase (AMPK, 1:1000, Cell Signaling Technology), phosphorylated (p-)AMPK (Thr172, 1:2000, Cell Signaling Technology), mammalian target of rapamycin (mTOR, 1:1000, Signalway Antibody), phospho(p-)mTOR (Ser2448, 1:1000, Cell Signaling Technology), p62 (1:400, Abcam), cathepsin D (CTSD, 1:500, Abcam), Bcl-2 (1:1000, Abcam), lysosomal-associated membrane protein 1 (LAMP1, 1:1000, Cell Signaling Technology), parkin (1:1000, Cell Signaling Technology), BCL-2/adenovirus E1B (19K)-interacting protein (BNIP3, 1:100, Cell Signaling Technology), cytochrome C (cyt c, 1:1000, Santa Cruz), VDAC1 (1:1000, Santa Cruz), COX-4 (1:1000, Abcam), and β-actin (1:5000, Abcam), followed by detection with an enhanced chemiluminescent substrate solution (Millipore, Watford, UK) after incubation with horseradish peroxidase-conjugated goat anti-rabbit or mouse IgG secondary antibody for 1 h at room temperature. The proteins were quantified by OD ratio using β-actin as a control. In case of mitochondria, COX-4 or VDAC1 was employed as the loading control. The extent of phosphorylation of each protein was evaluated with respect to the abundance of the native form (i.e., p-AKT/AKT, p-AMPK/AMPK, and p-mTOR/mTOR).

### Immunofluorescence

Briefly, paraffin-embedded sections were deparaffinized and washed three times with PBS for 5 min. Subsequently, the sections were immersed in EDTA-Tris solution (pH 9.0) for 30 min at 98 °C for antigen retrieval and rinsed three times with PBS for 5 min. Then, the slides were incubated with 10% non-immune goat serum for 30 min at room temperature to block non-specific staining. After this, the slides were incubated with primary antibodies: rabbit anti-LC3 (1:100, Abgent) and rabbit anti-LAMP1 (1:200, Cell Signaling Technology) in humidified chambers at 4 °C overnight. The next day, after washing these sections in PBS, isothiocyanate (TRITC)-conjugated anti-rabbit secondary antibodies (1:200, Santa Cruz) were then applied for 1 h at 37 °C. All stained specimens were observed under a confocal laser scanning microscope (Leica, Wetzlar, Germany). For the quantitative analysis, the average score of six randomly selected areas (three slides for each brain) was calculated using National Institutes of Health (NIH) Image Pro Plus 6.0 software.

### Immunoprecipitation

Briefly, homogenates (200 µg) were incubated with anti-Bcl-2 antibody (3 µg, Cell Signaling Technology, Beverly, MA, USA) at 4 °C overnight. Protein G-Sepharose beads (30 µl/tube, Sigma, St. Louis, MO, USA) were prewashed three times in immunoprecipitation (IP) buffer (10 mM Tris-Cl, pH 7.5, 150 mM sodium chloride, 2 mM EDTA, 0.5% Triton-100) for 15 min and incubated with a protein/antibody mixture under constant rotation at 4 °C for 2 h. The precipitant was collected by centrifugation at 10000 × g for 1 min and washed three times with IP buffer to remove nonspecifically bound proteins. Then, the immune-complexed beads, which were resuspended in SDS-PAGE loading buffer (60 µl/tube) and heated at 95 °C for 5 min, were removed by centrifugation at 10000 × *g*, and the supernatants were used for immunoblot detection of beclin-1 and Bcl-2. The left homogenates without IP were treated as the input controls.

### Statistical analysis

The data are reported as mean ± standard error of the mean (SEM). Statistical analyses were performed by one-way ANOVA, followed by Dunnett’s test as the post-hoc analysis. *P*-values of less than 0.05 were used as the criterion for significance in all types of data analyses.

## Results

### URB inhibits CCH-induced abnormal excessive autophagy in rats

**Fig. 1:**
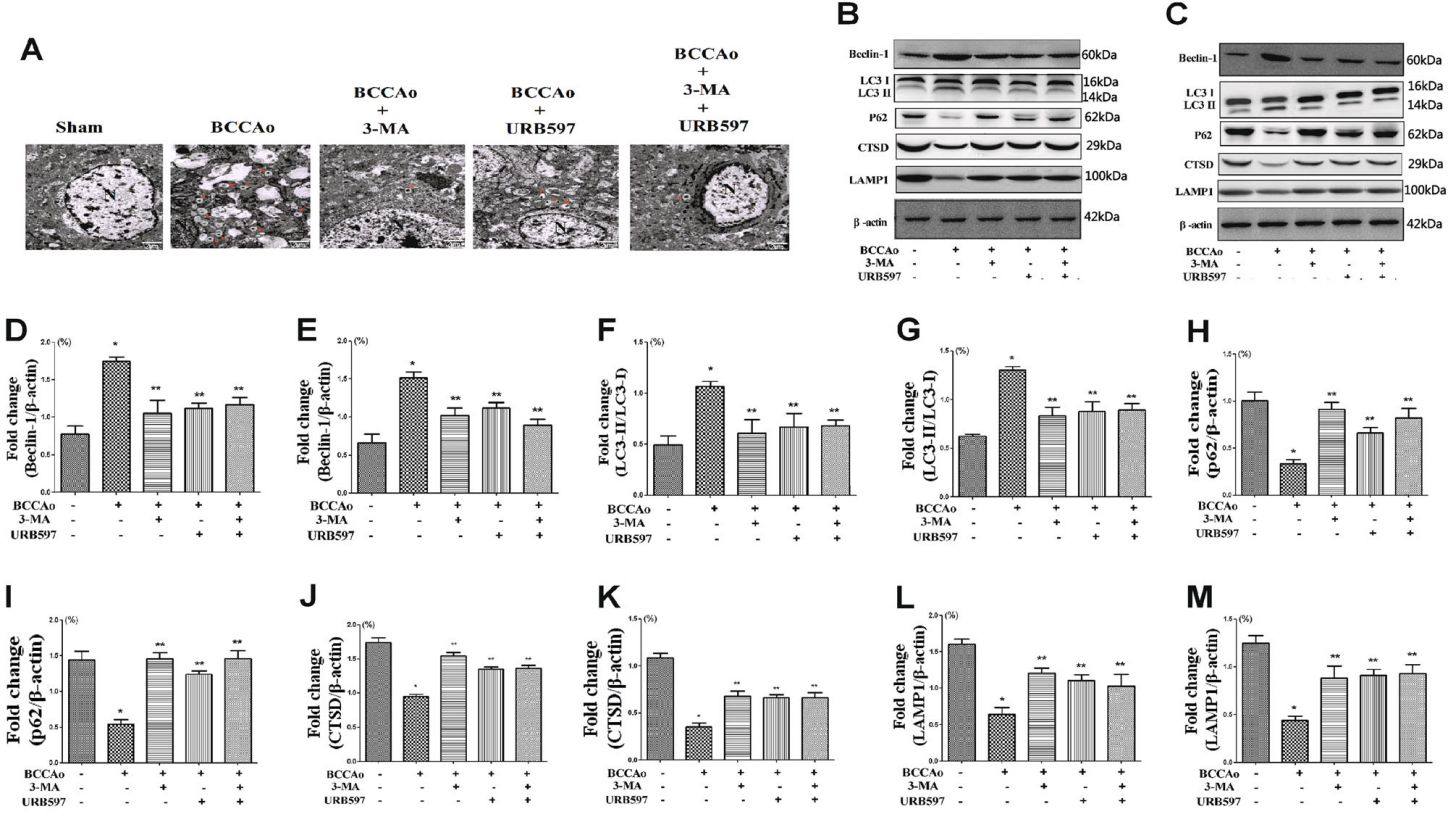
Effects of URB on CCH-induced autophagy. **a** Representative electron micrographs of autophagosomes in neurons (scale bars = 2 µm). N nucleus; autophagosomes are indicated by red arrows. **b**, **c** Representative western blot for Beclin-1, LC3, p62, CTSD, LAMP1, and β-actin in frontal cortex and hippocampus, respectively. **d**, **e** Relative densitometric density analysis of Beclin-1 protein bands (Beclin-1/β-actin) in frontal cortex and hippocampus, respectively. **f**, **g** Relative densitometric density analysis of LC3 protein bands (LC3-II/LC3-I) in frontal cortex and hippocampus, respectively. **h**, **i** Relative densitometric density analysis of p62 protein bands (p62/β-actin) in frontal cortex and hippocampus, respectively. **j**, **k** Relative densitometric density analysis of CTSD protein bands (CTSD/β-actin) in frontal cortex and hippocampus, respectively. **l**, **m** Relative densitometric density analysis of LAMP1 protein bands (LAMP1/β-actin) in frontal cortex and hippocampus, respectively. Data are expressed as mean ± SEM (*n* = 5). *P < 0.05 versus sham group. **P < 0.05 versus BCCAo group

To investigate whether CCH promotes the excessive autophagic activities and the excessive autophagic activities are altered in neurons after URB treatment, brain sections were examined by electron microscopy. Compared with controls, autophagosomes were remarkably found in BCCAo group. Nevertheless, the autophagy inhibitor 3-MA or URB could significantly decrease the number of autophagosomes (Fig. 1a).

**Fig. 2:**
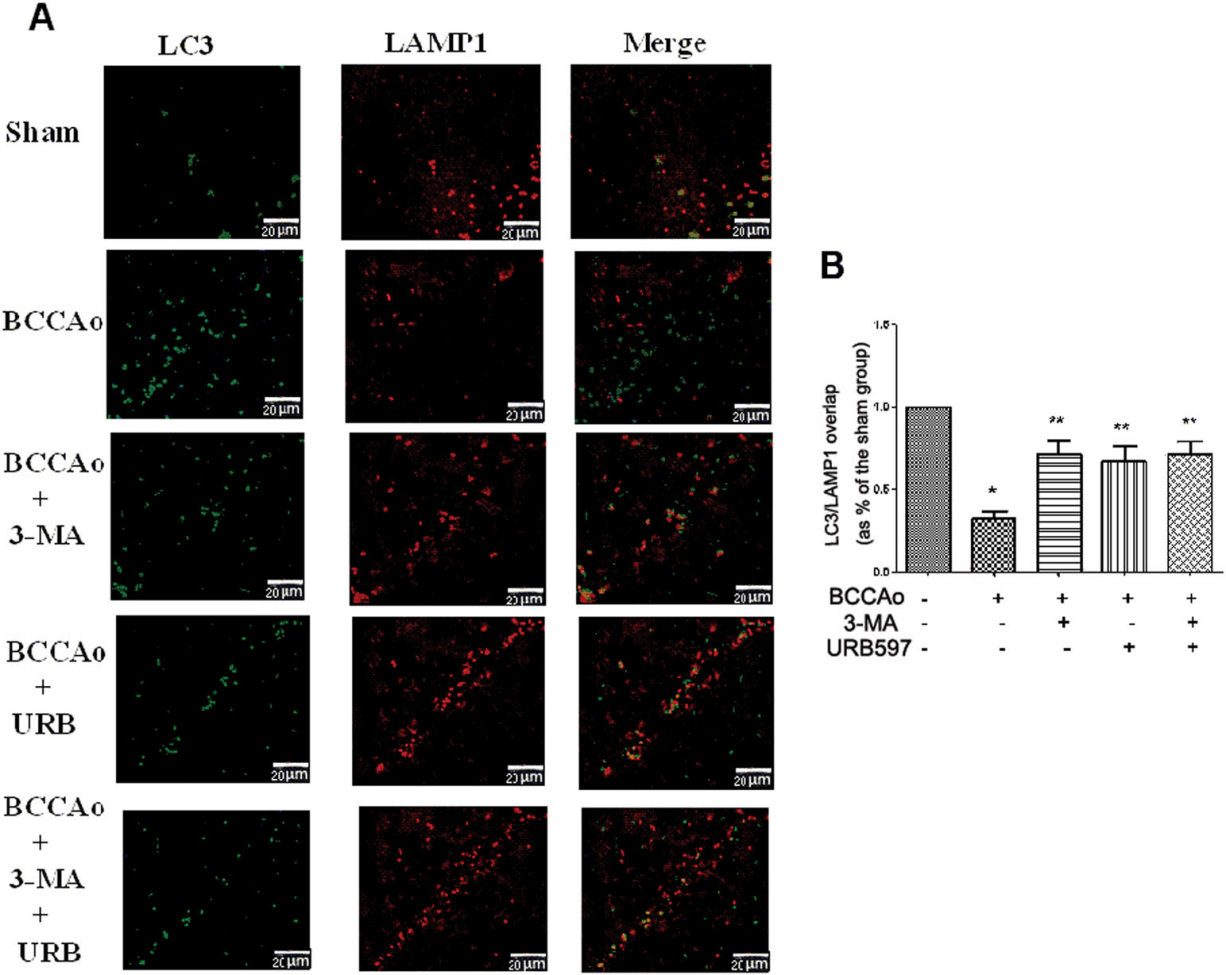
Effects of URB on CCH-induced inhibition of autophagosome–lysosome fusion. **a** Representative immunofluorescence staining for LC3 and LAMP1 in neurons (scale bars = 20 µm). Autophagosome: green LC3-positive puncta; lysosome: red LAMP1-positive puncta; autophagosome–lysosome fusion: yellow LC3/LAMP1 overlap. Depletion of LC3 and LAMP1 double-positive punctas and single LAMP1-positive punctas are apparent after CCH. **b** Quantification of LC3/LAMP1 colocalization co-efficiency (ratio of Sham group). The LC3/LAMP1 overlap in Sham group is set to 1. *P < 0.05 versus sham group. **P < 0.05 versus BCCAo group

To further determine whether the process of autophagy is involved in the neuroprotective effect of URB under the CCH condition, the autophagy-associated proteins such as LC3, beclin-1, and p62 and lysosome-related proteins such as CTSD and LAMP1 were assessed in hippocampus and frontal cortex. CCH significantly decreased the protein expression of p62, CTSD, and LAMP1 and increased the protein expression of beclin-1 and the LC3-II to LC3-I ratio in frontal cortex and hippocampus relative to sham-operated animals. In addition, we examined the colocalization of lysosomes and autophagosomes after CCH. The fluorescent LC3-positive autophagosomes were easily observed in BCCAo rats, and did not colocalize with LAMP1-positive lysosomes. The above results showed that a backlog of unfused autophagosomes can not be efficiently processed by lysosomes, leading to autophagosomes accumulation. However, 3-MA or URB could significantly reverse these results (Fig. 1b, c and Fig. 2), suggesting that URB may exert the neuroprotective effect through the inhibition of abnormal excessive autophagy.

### URB alleviates CCH-induced hippocampal synaptic degradation in rats

**Fig. 3:**
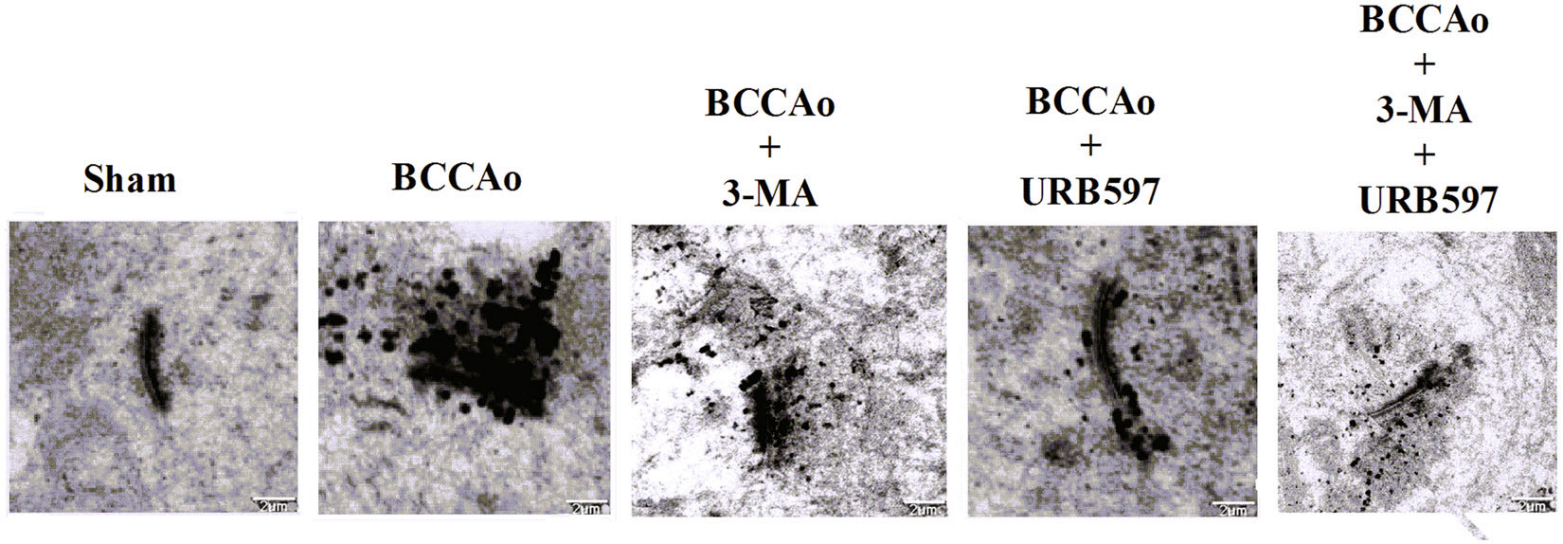
Effects of URB on CCH-induced alterations of PSDs ultrastructure. Representative electron micrographs of the PSDs ultrastructure in neurons (scale bars = 2 µm)

Considering that autophagosomes are formed in dendrites and synaptic terminal regions, we hypothesize that accumulated autophagosomes may result in decreased synaptic plasticity, which has been confirmed in our previous study^7^. Hence, the ultrastructural changes of postsynaptic densities (PSDs) were examined by electron microscopy. PSDs from the hippocampal CA1 regions in the control rats were thin and curved, whereas PSDs in the CCH rats became thick and straight with high deposition of EPTA-stained proteins. However, these phenomena were significantly changed after treatment with 3-MA, demonstrating that abnormal excessive autophagy might induce hippocampal synaptic damage. Furthermore, PSDs ultrastructure recovered curved with clear layers and the deposition of EPTA-stained proteins was significantly decreased after treatment with URB (Fig. 3). These results indicated that excessive autophagy may exacerbate synaptic degradation.

### URB attenuates CCH-induced mitochondrial dysfunction and mitophagy in rats

**Fig. 4:**
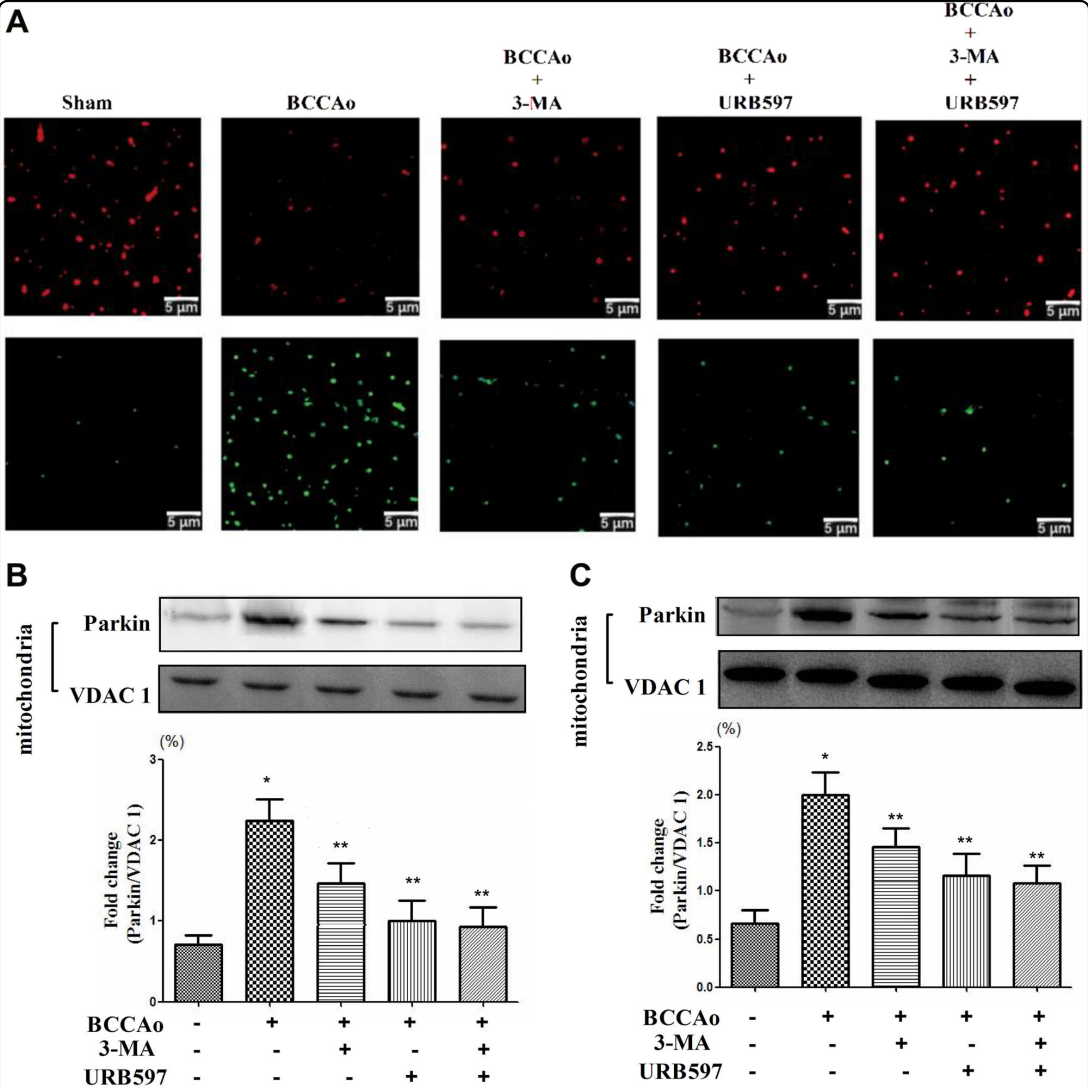

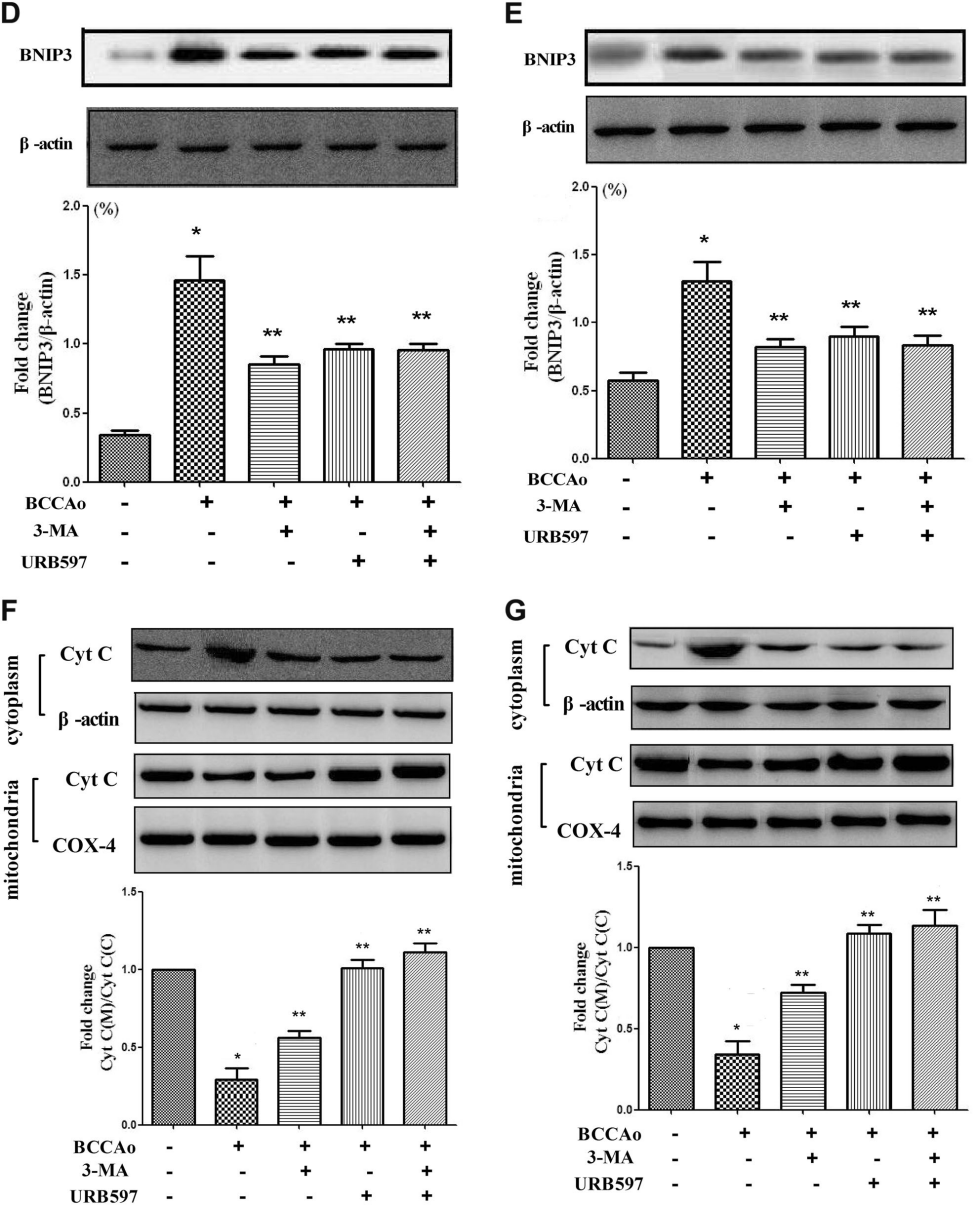
(Continued)

Since it is well established that mitochondrial dysfunction contributes to autophagy induction^22,23^, we examined whether mitochondrial damage and mitophagy were launched by CCH and whether URB protected against mitochondrial damage and mitophagy. JC-1 staining was used to confirm whether URB treatment reversed the mitochondrial dysfunction. Mitochondrial depolarization is indicated by a decrease in the JC-1 red/green fluorescence intensity ratio. CCH significantly reduced the red/green fluorescence intensity ratio as compared with the control group. Nevertheless, treatment with 3-MA or URB abrogated the CCH-induced decreased red/green fluorescence intensity ratio (Fig. 4a). These results suggest that CCH promotes mitochondrial dysfunction, and this is relieved by URB.

To further investigate whether there is a link between mitochondrial dysfunction and autophagy, we examined the level of parkin which is a marker protein of receptor-independent mitophagy and plays a important role in mitochondrial fragmentation and mitophagy during cell death^24,25^. The level of mitochondrial parkin was significantly increased by CCH in frontal cortex and hippocampus, but this increase was attenuated by URB treatment (Fig. 4b, c). Furthermore, the level of BNIP3, which is involved in the receptor-dependent mitophagy (such as BNIP3-cyt C-related mitophagy)^26^, was significantly increased in the frontal cortex and hippocampus following CCH, which induced the level of cyc C remarkably decreased in mitochondria following CCH in the frontal cortex and hippocampus, indicating that cyt C is released from the mitochondria to the cytosol during mitochondrial dysfunction. However, treatment with 3-MA or URB reversed this tendency (Fig. 4d–g). URB inhibited the loss of mitochondrial function, the increase of parkin and BNIP3, and the release of cyt C, demonstrating its protective activity on CCH-induced mitochondrial dysfunction and mitophagy.

### AKT/mTOR pathway, but not AMPK/mTOR pathway, promotes mTOR phosphorylation after URB treatment

**Fig. 5:**
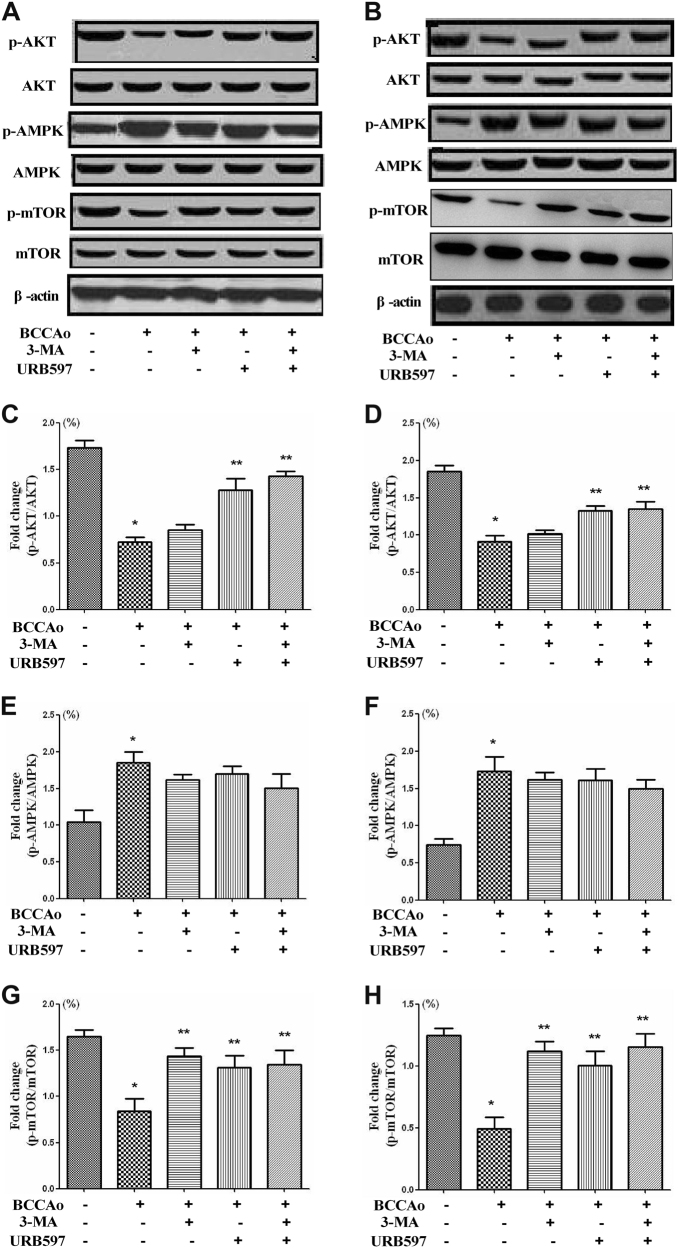
Effects of URB on the AKT/mTOR and AMPK/mTOR signaling after CCH. **a**, **b** Representative western blot for p-AKT, AKT, p-AMPK, AMPK, p-mTOR, mTOR, and β-actin in frontal cortex and hippocampus, respectively. **c**, **d** Relative densitometric density analysis of the p-AKT protein bands (p-AKT/AKT) in frontal cortex and hippocampus, respectively. **e**, **f** Relative densitometric density analysis of the p-AMPK protein bands (p-AMPK/AMPK) in frontal cortex and hippocampus, respectively. **g**, **h** Relative densitometric density analysis of the p-mTOR protein bands (p-mTOR/mTOR) in frontal cortex and hippocampus, respectively. Data are expressed as mean ± SEM (*n* = 5). *P < 0.05 versus sham group. **P < 0.05 versus BCCAo group

To explore the possible mechanisms that could inhibit abnormal excessive autophagy after URB treatment, we examined AKT/mTOR and AMPK/mTOR signaling pathways in frontal cortex and hippocampus, which were the important regulators of the autophagic process^27^. CCH increased p-AMPK expression and decreased p-AKT and p-mTOR levels. URB treatment significantly increased p-AKT and p-mTOR levels, while p-AMPK expression was unaltered (Fig. 5). These results indicate that URB may suppress abnormal excessive autophagy induced by CCH in part by promoting AKT/mTOR pathway.

### URB inhibits CCH-induced dissociation of beclin-1/Bcl-2 complex

**Fig. 6:**
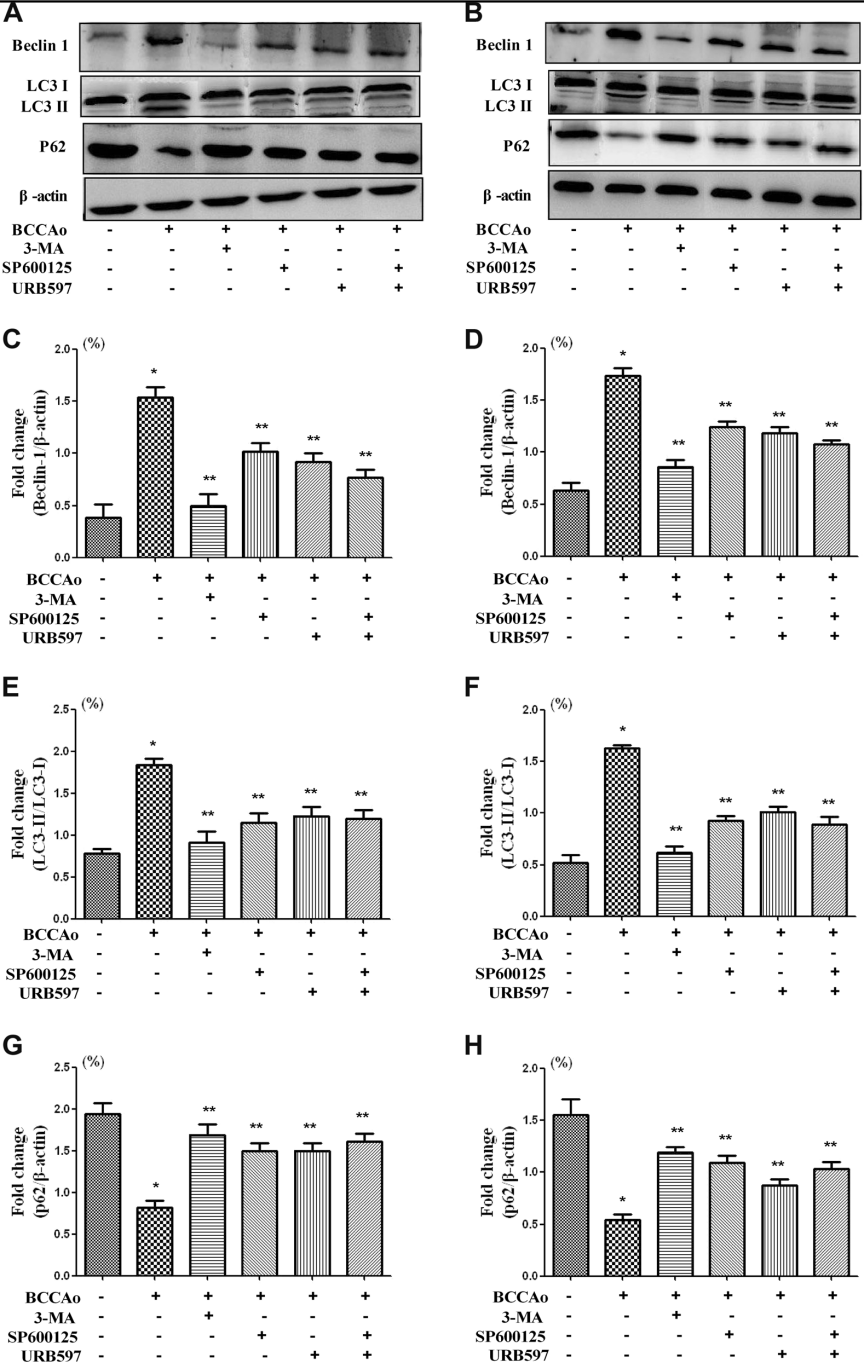
Involvement of JNK-dependent non-nuclear pathway in autophagy following CCH. **a**, **b** Representative western blot for Beclin-1, LC3, p62, and β-actin in frontal cortex and hippocampus, respectively. **c**, **d** Relative densitometric density analysis of Beclin-1 protein bands (Beclin-1/β-actin) in frontal cortex and hippocampus, respectively. **e**, **f** Relative densitometric density analysis of LC3 protein bands (LC3-II/LC3-I) in frontal cortex and hippocampus, respectively. **g**, **h** Relative densitometric density analysis of p62 protein bands (p62/β-actin) in frontal cortex and hippocampus, respectively. Data are expressed as mean ± SEM (*n* = 5). *P < 0.05 versus sham group. **P < 0.05 versus BCCAo group

In our previous study^8^, JNK-dependent signaling was activated to promote neuronal apoptosis under CCH condition, which could be inhibited by URB treatment. Interestingly, according to Guo et al.^28^, 3-MA decreased the level of p-JNK, which may act like the JNK inhibitor SP600125. Hence, we hypothesize that SP600125 may also act in part like 3-MA. To investigate whether SP600125 could inhibit CCH-induced abnormal excessive autophagy, the protein levels of beclin-1, p62, and LC3 were assessed in the hippocampus and frontal cortex. Similar to 3-MA, SP600125 significantly increased the protein expression of p62 and decreased the protein expression of beclin-1 and the LC3-II to LC3-I ratio relative to BCCAo rats (Fig.6), confirming that the non-nuclear JNK pathway may be involved in the process of CCH-induced abnormal excessive autophagy. URB may suppress abnormal excessive autophagy induced by CCH through the inhibition of JNK-dependent signaling.

**Fig. 7:**
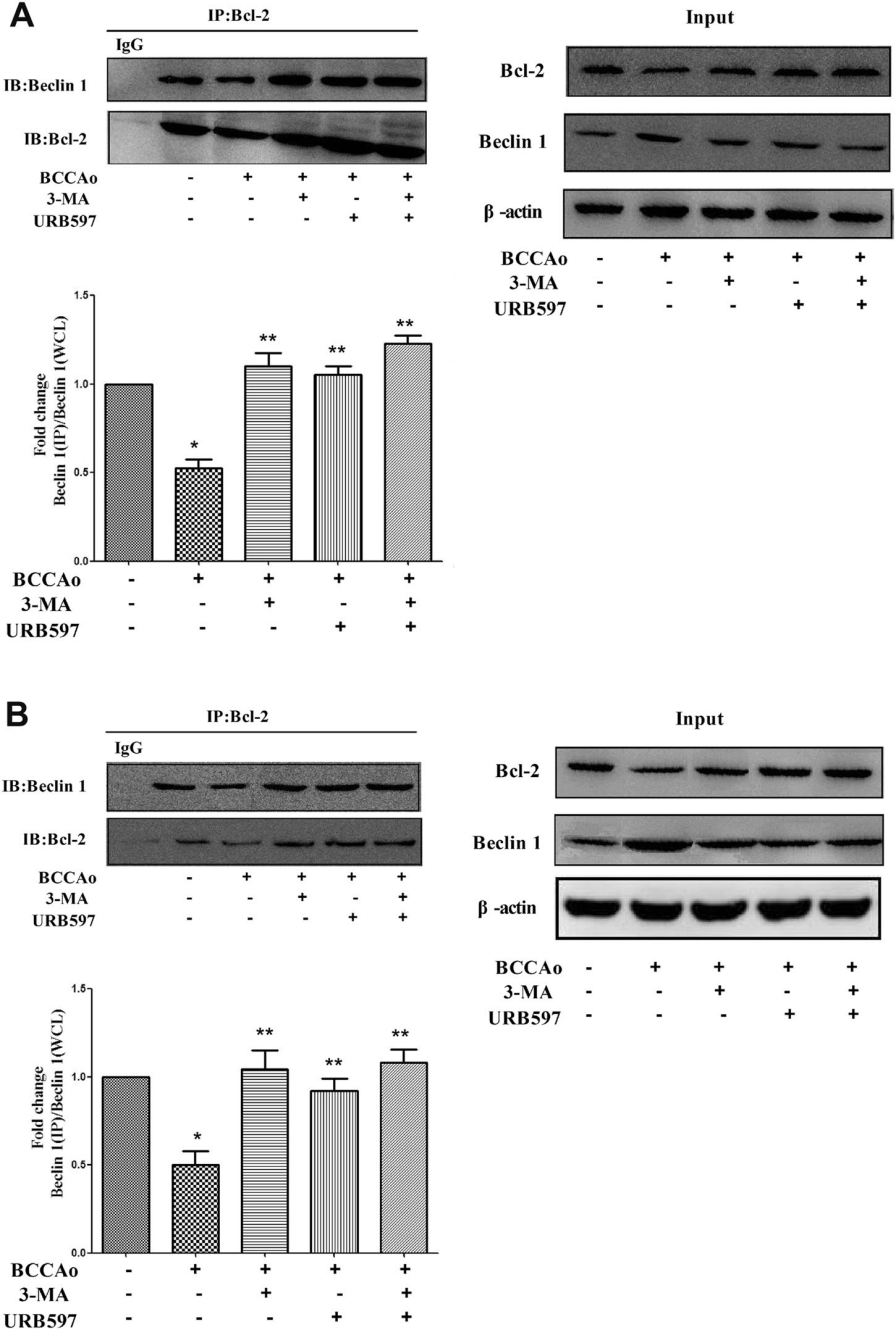
Effects of URB on the Bcl-2/Beclin 1 complex alterations after CCH. **a**, **b** Representative immunoprecipitation and immunoblot for Bcl-2, Beclin 1, and β-actin in frontal cortex and hippocampus. The relative abundance of beclin 1 in IP complex was determined by western blotting, and was compared to the level of beclin 1 in whole-cell lysates (WCL). The result suggested that URB inhibited the dissociation of beclin 1 from Bcl-2. Data are expressed as mean ± SEM (*n* = 5). *P < 0.05 versus sham group. **P < 0.05 versus BCCAo group

To further explore the possible mechanisms that could inhibit abnormal excessive autophagy through the inhibition of JNK-dependent Bcl-2 signaling after URB treatment in hippocampus and frontal cortex. The binding of Bcl-2 with beclin-1 into a complex was measured using IP with a Bcl-2 antibody (Fig. 7). The relative abundance of beclin-1 in Bcl-2/beclin-1 complex was determined by western blotting, and was compared to the level of beclin-1 in whole-cell lysates (WCL). Compared with CCH, 3-MA or URB could significantly increase the level of beclin-1 in IP complex/beclin-1 in WCL. The result showed that URB inhibited the dissociation of beclin-1 from Bcl-2, leading to the suppression of abnormal excessive autophagy.

## Discussion

In neurons, autophagy at a low level is important for maintaining homeostasis and protein quality under normal conditions. Although autophagy is enhanced following cerebral ischemia, the effect of high level of autophagy is controversial, and depends on brain maturity, region, insult severity, and stage of ischemia^29^. In our present study, at the stage of CCH, although autophagy was enhanced, lysosomal malfunction contributed to disruption of these enhanced autophagy. Hence, the role of enhanced autophagy under CCH condition was destructive. We call this impaired process of autophagy with high level of autophagic expression as abnormal excessive autophagy. Furthermore, to our knowledge, we confirmed for the first time that mitophagy was launched with the development of neuronal apoptosis under the condition of CCH by both BNIP3-cyt C-related pathway and parkin-mediated signaling. In addition, we firstly demonstrated that URB may alleviate the abnormal excessive autophagy by inhibiting the disruption of beclin-1/Bcl-2 pathway. In our opinion, we suggest that URB should be used when the risk of CCH is established and be expected to continue use until the end of CCH phase.

The process of autophagy mainly consists of three sequential steps including sequestration, transportation, and degradation^30^. At the initial stage, consistent with other studies^31,32^, CCH promoted autophagy through overexpression of beclin-1 and LC-3 II. Excessive autophagic initiation may be associated with neuronal damage^32^. At the stage of transportation and degradation, one recent study reported that the levels of p62 increased beginning at 2 weeks in the cortex and at 4 weeks in the hippocampus and remained elevated throughout the study period. P62 is the receptor protein which delivers the injured organelles and potentially toxic protein aggregates to autophagosomes^33,34^. Stimulation of autophagy flux causes depletion of p62 along with other autophagic substrates. The increase of p62 suggests that autophagic clearance and transportation is impaired^35^. Interestingly, in our present study, we found the level of p62 was decreased after CCH, indicating that autophagic turnover and transportation was not impaired. At the stage of degradation, autophagosomes and their cargo are degraded within lysosomes by lysosomal hydrolases^36^. Lysosomal function was further investigated in our study. The levels of the soluble lysosomal enzyme CTSD and lysosomal membrane proteins LAMP1 were decreased after CCH, indicating that lysosomal malfunction may contribute to the autophagosomes accumulation. In our opinion, we treat the accumulation of autophagosomes as abnormal excessive autophagy. Taken together, our data revealed that the abnormal excessive autophagy may be a potential mechanism contributing to neuronal cell death due to CCH, which could be the possible therapeutic target for our research. Subsequently, we found that the autophagy inhibitor 3-MA or URB decreased the autophagosomes accumulation, the expression of beclin-1, and the LC3-II to LC3-I ratio and increased the levels of p62, CTSD, and LAMP1, suggesting that URB may inhibit the lysosomal dysfunction after CCH, which could activate its neuroprotective effects against the abnormal excessive autophagy.

The PSDs is a specialized cytoskeletal structure lying beneath the postsynaptic membrane. Neurotransmitter receptors, ion channels, and signaling molecules are highly enriched in PSDs, suggesting an important function for PSDs in the anchoring and targeting of functional proteins required for receiving and transducing synaptic signal in postsynaptic neurons^37^. CCH might lead to the accumulation of ubiquitinated proteins in rat hippocampal CA1 PSDs, resulting in the impairment of synaptic plasticity and cognitive decline^7,38^. In the present study, to confirm whether there is a link between ubiquitinated proteins aggregates and the accumulation of autophagosomes, the ultrastructural changes of PSDs were examined by EPTA electron microscopy. We found a great amount of ubiquitinated proteins accumulated in PSDs after CCH. This phenomenon could be significantly reduced by 3-MA, indicating that abnormal excessive autophagy may contribute to the accumulation of ubiquitinated proteins, which is consistent with another study^39^. In our opinion, we firstly proposed that accumulation of autophagosomes—ubiquitinated proteins aggregates in PSDs—the impairment of synaptic plasticity—cognitive impairment—might be one of the possible mechanisms involved in the effect of autophagy on cognitive impairment. Compared to BCCAo, URB could significantly decrease the accumulation of ubiquitinated proteins, suggesting the neuroprotective effects against the abnormal excessive autophagy.

Mitophagy, a selective pattern of autophagy, eliminates dysfunctional mitochondria under normal as well as pathological conditions including cerebral ischemia^40^. To our knowledge, only one published research showed that inefficient BNIP3-mediated mitophagy may constitute mechanisms underlying neuronal cell damage during CCH in vitro^26^. The underlying mechanisms of mitophagy under the CCH condition are not well understood yet. According to our mitochondrial functional test, mitochondrial dysfunction, which has been proposed as the contributor to mitophagy^22^, was induced by CCH. However, 3-MA could significantly reverse this phenomenon, suggesting that the abnormal excessive autophagy might in turn aggravate the mitochondrial dysfunction. We further investigated whether two basic pathways of mitophagy including receptor-dependent signaling (such as BNIP3-cyt C-related mitophagy) and receptor-independent mechanism (parkin-mediated mitophagy) were invloved in the process of mitophagy under CCH. We found that CCH induced the increase of parkin and BNIP3 and the release of cyt C from mitochondria to cytosol, which was significantly attenuated by 3-MA treatment, indicating that mitophagy was launched in part by the two basic pathways under CCH condition. Furthermore, URB could demonstrate the similar effects with 3-MA, suggesting that URB may inhibit the abnormal excessive mitophagy following CCH through both BNIP3-cyt C-related pathway and parkin-mediated signaling.

The correlation between apoptosis and autophagy and their pathologic processes in CCH are not clear. According to our previous and present studies [8], under the condition of CCH, neuronal apoptosis and autophagy were all enhanced. The activated pathways of autophagy and apoptosis may share some common components. We found that JNK-dependent Bcl-2 signaling was activated in neuronal apoptosis after CCH^8^. Another study reported that 3-MA decreased the level of p-JNK^28^, which may play the role of JNK inhibitor SP600125. Consistent with one study^41^, we found that SP600125 significantly increased the protein expression of p62 and decreased the protein expression of beclin-1 and the LC3-II to LC3-I ratio, indicating that the non-nuclear JNK pathway may be involved in the process of CCH-induced abnormal excessive autophagy. Given that JNK activation is the common pathway in neuronal apoptosis and autophagy, we speculate neuronal apoptosis and autophagy are mutually reinforcing, resulting in the neuronal damage following CCH. Beclin-1 possesses a BH3 domain that makes its interaction with the BH3 receptor domain of anti-apoptotic proteins of the Bcl-2 family. Evidence showed that autophagy could be launched by disrupting the beclin-1/Bcl-2 complex^42^. We also found that the protein level of Bcl-2 was significantly decreased under CCH condition^8^. However, to our knowledge, beclin-1/Bcl-2 signaling following CCH is not elucidated yet. In present study, we found that CCH promoted beclin-1 from beclin-1/Bcl-2 complex to cell fluid, leading to the induction of autophagy. However, 3-MA or URB inhibited the dissociation of beclin-1 from Bcl-2, suggesting that UBR may suppress abnormal excessive autophagy after CCH by turning off the autophagy switch of the beclin-1/Bcl-2 complex. Taken together, JNK-dependent Bcl-2 signaling and beclin-1/Bcl-2 complex were involved in the neuroprotective effect of URB.

In conclusion, our present data may provide new insights into the roles of autophagy and mitophagy in the process of CCH-induced neuronal death. CCH induced the lysosomal dysfunction which may promote the accumulation of autophagosomes, resulting in the abnormal excessive autophagy. Furthermore, abnormal excessive autophagy may contribute to the accumulation of ubiquitinated proteins in PSDs, leading to the impairment of synaptic plasticity and cognitive impairment. In addition, BNIP3-cyt C- and parkin-mediated mitophagy may be two possible mechanisms underlying neuronal death following CCH. Besides CCH-induced neuronal apoptosis, JNK pathway may also be involved in the process of CCH-induced autophagy. However, URB could inhibit impaired autophagy degradation and the disruption of beclin-1/ Bcl-2 complex and subsequently cut off BNIP3-cyt C- and parkin-required mitophagy, finally preventing the abnormal excessive autophagy and mitophagy. These findings might extend previous studies suggesting a beneficial role for URB in brain chronic ischemic injury.

## References

[1] Su SH (2014). Cognitive function, depression, anxiety and quality of life in Chinese patients with untreated unruptured intracranial aneurysms. J. Clin. Neurosci..

[2] Su SH, Hai J, Zhang L, Yu F, Wu YF (2013). Assessment of cognitive function in adult patients with hemorrhagic moyamoya disease who received no surgical revascularization. Eur. J. Neurol..

[3] Aliev G (2008). Atherosclerotic lesions and mitochondria DNA deletions in brain microvessels: implication in the pathogenesis of Alzheimer’s disease. Vasc. Health Risk Manag..

[4] Hainsworth AH, Markus HS (2008). Do in vivo experimental models reflect human cerebral small vessel disease? A systematic review. J. Cereb. Blood Flow Metab..

[5] Sekhon LH, Spence I, Morgan MK, Weber NC (1998). Long-term potentiation saturation in chronic cerebral hypoperfusion. J. Clin. Neurosci..

[6] Su SH, Wu YF, Lin Q, Hai J (2017). Cannabinoid receptor agonist WIN55,212-2 and fatty acid amide hydrolase inhibitor URB597 ameliorate neuroinflammatory responses in chronic cerebral hypoperfusion model by blocking NF-κB pathways. Naunyn Schmiedebergs Arch. Pharmacol..

[7] Su SH (2016). Cannabinoid receptor agonist WIN55,212-2 and fatty acid amide hydrolase inhibitor URB597 may protect against cognitive impairment in rats of chronic cerebral hypoperfusion via PI3K/AKT signaling. Behav. Brain Res..

[8] Su SH, Wu YF, Lin Q, Yu F, Hai J (2015). Cannabinoid receptor agonist WIN55,212-2 and fatty acid amide hydrolase inhibitor URB597 suppress chronic cerebral hypoperfusion-induced neuronal apoptosis by inhibiting c-Jun N-terminal kinase signaling. Neuroscience.

[9] Smith ML, Auer RN, Siesjö BK (1984). The density and distribution of ischemic brain injury in the rat following 2-10min of forebrain ischemia. Acta Neuropathol..

[10] Choi BR (2011). Synergistic memory impairment through the interaction of chronic cerebral hypoperfusion and amlyloid toxicity in a rat model. Stroke.

[11] Choi AM, Ryter SW, Levine B (2013). Autophagy in human health and disease. N. Engl. J. Med..

[12] Rami A, Langhagen A, Steiger S (2008). Focal cerebral ischemia induces upregulation of Beclin 1 and autophagy-like cell death. Neurobiol. Dis..

[13] Liu L (2015). Baclofen mediates neuroprotection on hippocampal CA1 pyramidal cells through the regulation of autophagy under chronic cerebral hypoperfusion. Sci. Rep..

[14] Qiu L (2016). Chronic cerebral hypoperfusion enhances Tau hyperphosphorylation and reduces autophagy in Alzheimer’s disease mice. Sci. Rep..

[15] Hu M (2017). Autophagy and Akt/CREB signalling play an important role in the neuroprotective effect of nimodipine in a rat model of vascular dementia. Behav. Brain. Res..

[16] Shao A (2016). Enhancement of autophagy by histone deacetylase inhibitor trichostatin A amelioratesneuronal apoptosis after subarachnoid hemorrhage in rats. Mol. Neurobiol..

[17] Paxinos, G. & Franklin, K. *The Mouse Brain In Stereotaxic Coordinates* 2nd edn (Academic Press Inc., San Diego, CA, 2001).

[18] Lu J, Wu DM, Zheng YL, Hu B, Zhang ZF (2010). Purple sweet potato color alleviates D-galactose-induced brain aging in old mice by promoting survival of neurons via PI3K pathway and inhibiting cytochrome C-mediated apoptosis. Brain Pathol..

[19] Farkas E, Luiten PG, Bari F (2007). Permanent, bilateral common carotid artery occlusion in the rat: a model for chronic cerebral hypoperfusion-related neurodegenerative diseases. Brain Res. Rev..

[20] Hu BR, Martone ME, Jones YZ, Liu CL (2000). Protein aggregation after transient cerebral ischemia. J. Neurosci..

[21] Wang D (2017). URB597 improves cognitive impairment induced by chronic cerebral hypoperfusion by inhibiting mTOR-dependent autophagy. Neuroscience.

[22] Vosler PS, Graham SH, Wechsler LR, Chen J (2009). Mitochondrial targets for stroke: focusing basic science research toward development of clinically translatable therapeutics. Stroke.

[23] Kubli DA, Gustafsson AB (2012). Mitochondria and mitophagy: the yin and yang of cell death control. Circ. Res..

[24] Springer W, Kahle PJ (2011). Regulation of PINK1-Parkin-mediated mitophagy. Autophagy.

[25] Ashrafi G, Schwarz TL (2013). The pathways of mitophagy for quality control and clearance of mitochondria. Cell Death Differ..

[26] Song Y (2018). Involvement of impaired autophagy and mitophagy in Neuro-2a cell damage under hypoxic and/or high-glucose conditions. Sci. Rep..

[27] Wang P (2012). Induction of autophagy contributes to the neuroprotection of nicotinamide phosphoribosyltransferase in cerebral ischemia. Autophagy.

[28] Guo Z (2014). A combination of four active compounds alleviates cerebral ischemia-reperfusion injury in correlation with inhibition of autophagy and modulation of AMPK/mTOR and JNK pathways. J. Neurosci. Res..

[29] Xu F, Gu JH, Qin ZH (2012). Neuronal autophagy in cerebral ischemia. Neurosci. Bull..

[30] Mizushima N (2007). Autophagy: process and function. Genes Dev..

[31] Yang Z (2014). Microglial activation with reduction in autophagy limits white matter lesions and improves cognitive defects during cerebral hypoperfusion. Curr. Neurovasc. Res..

[32] Zou W (2018). The role of autophagy in the correlation between neuron damage and cognitive impairment in rat chronic cerebral hypoperfusion. Mol. Neurobiol..

[33] Bjorkoy G, Lamark T, Johansen T (2006). p62/SQSTM1: a missing link between protein aggregates and the autophagy machinery. Autophagy.

[34] Pankiv S (2007). p62/SQSTM1 binds directly to Atg8/LC3 to facilitate degradation of ubiquitinated protein aggregates by autophagy. J. Biol. Chem..

[35] Coffey EE, Beckel JM, Laties AM, Mitchell CH (2014). Lysosomal alkalization and dysfunction in human fibroblasts with the Alzheimer’s disease-linked presenilin 1 A246E mutation can be reversed with cAMP. Neuroscience.

[36] Mizushima N, Levine B, Cuervo AM, Klionsky DJ (2008). Autophagy fights disease through cellular self-digestion. Nature.

[37] Kennedy MB (1994). The biochemistry of synaptic regulation in the central nervous system. Annu. Rev. Biochem..

[38] Hai J (2011). Chronic cerebral hypoperfusion in rats causes proteasome dysfunction and aggregation of ubiquitinated proteins. Brain Res..

[39] Sarkar C (2014). Impaired autophagy flux is associated with neuronal cell death after traumatic brain injury. Autophagy.

[40] Gabryel B, Kost A, Kasprowska D (2012). Neuronal autophagy in cerebral ischemia–a potential target for neuroprotective strategies?. Pharmacol. Rep..

[41] Ren H (2010). DJ-1, a cancer and Parkinson’s disease associated protein, regulates autophagy through JNK pathway in cancer cells. Cancer Lett..

[42] Qi Z (2015). Bcl-2 phosphorylation triggers autophagy switch and reduces mitochondrial damage in limbremote ischemic conditioned rats after ischemic stroke. Transl. Stroke Res..

